# Bakuchiol Attenuates Oxidative Stress and Neuron Damage by Regulating Trx1/TXNIP and the Phosphorylation of AMPK After Subarachnoid Hemorrhage in Mice

**DOI:** 10.3389/fphar.2020.00712

**Published:** 2020-05-15

**Authors:** Haixiao Liu, Wei Guo, Hao Guo, Lei Zhao, Liang Yue, Xia Li, Dayun Feng, Jianing Luo, Xun Wu, Wenxing Cui, Yan Qu

**Affiliations:** ^1^Department of Neurosurgery, Tangdu Hospital, The Fourth Military Medical University, Xi’an, China; ^2^Department of Pathology, University of Texas Southwestern Medical Center, Dallas, TX, United States

**Keywords:** bakuchiol, subarachnoid hemorrhage, early brain injury, oxidative stress, apoptosis, thioredoxin, AMP-activated protein kinase

## Abstract

Subarachnoid hemorrhage (SAH) is a fatal cerebrovascular condition with complex pathophysiology that reduces brain perfusion and causes cerebral functional impairments. An increasing number of studies indicate that early brain injury (EBI), which occurs within the first 72 h of SAH, plays a crucial role in the poor prognosis of SAH. Bakuchiol (Bak) has been demonstrated to have multiorgan protective effects owing to its antioxidative and anti-inflammatory properties. The present study was designed to investigate the effects of Bak on EBI after SAH and its underlying mechanisms. In this study, 428 adult male C57BL/6J mice weighing 20 to 25 g were observed to investigate the effects of Bak administration in an SAH animal model. The neurological function and brain edema were assessed. Content of MDA/3-NT/8-OHdG/superoxide anion and the activity of SOD and GSH-Px were tested. The function of the blood-brain barrier (BBB) and the protein levels of claudin-5, occludin, zonula occludens-1, and matrix metalloproteinase-9 were observed. TUNEL staining and Fluoro-Jade C staining were conducted to evaluate the death of neurons. Ultrastructural changes of the neurons were observed under the transmission electron microscope. Finally, the roles of Trx, TXNIP, and AMPK in the protective effect of Bak were investigated. The data showed that Bak administration 1) increased the survival rate and alleviated neurological functional deficits; 2) alleviated BBB disruption and brain edema; 3) attenuated oxidative stress by reducing reactive oxygen species, MDA, 3-NT, 8-OHdG, gp91^phox^, and 4-HNE; increased the activities of SOD and GSH-Px; and alleviated the damage to the ultrastructure of mitochondria; 4) inhibited cellular apoptosis by regulating the protein levels of Bcl-2, Bax, and cleaved caspase-3; and 5) upregulated the protein levels of Trx1 as well as the phosphorylation of AMPK and downregulated the protein levels of TXNIP. Moreover, the protective effects of Bak were partially reversed by PX-12 and compound C. To summarize, Bak attenuates EBI after SAH by alleviating BBB disruption, oxidative stress, and apoptosis *via* regulating Trx1/TXNIP expression and the phosphorylation of AMPK. Its powerful protective effects might make Bak a promising novel drug for the treatment of EBI after SAH.

## Introduction

Although the treatment of subarachnoid hemorrhage (SAH), a severe subtype of stroke, has been discussed a lot in recent years, the mortality and morbidity of SAH remain high and it leads to the loss of many years of productive life (Macdonald and Schweizer, 2017). The loss of neurological function results from the primary injury directly caused by the hemorrhage and secondary injury following the primary injury (Macdonald, 2014).

Recently, an increasing number of studies have indicated that early brain injury (EBI), which occurs within the first 72 h of SAH, plays a crucial role in the poor prognosis of SAH (Liu L. et al., 2017; Macdonald and Schweizer, 2017). EBI is the primary cause of SAH-associated histological injuries, function deficits, and death (Sehba et al., 2012). Therefore, the targeting of EBI might be the most effective method for the treatment of SAH. During EBI, hemorrhage causes many pathophysiology problems, including the increase in intracranial pressure, the decrease in cerebral blood flow, and the global cerebral ischemia (Fujii et al., 2013). A complex mechanism, including blood-brain barrier (BBB) disruption, neuroinflammation, oxidative stress, and neuronal apoptosis is involved in the process of injury after SAH, which ultimately leads to cell death and severe damage to neurological functions (Lucke-Wold et al., 2016).

**Figure 1:**
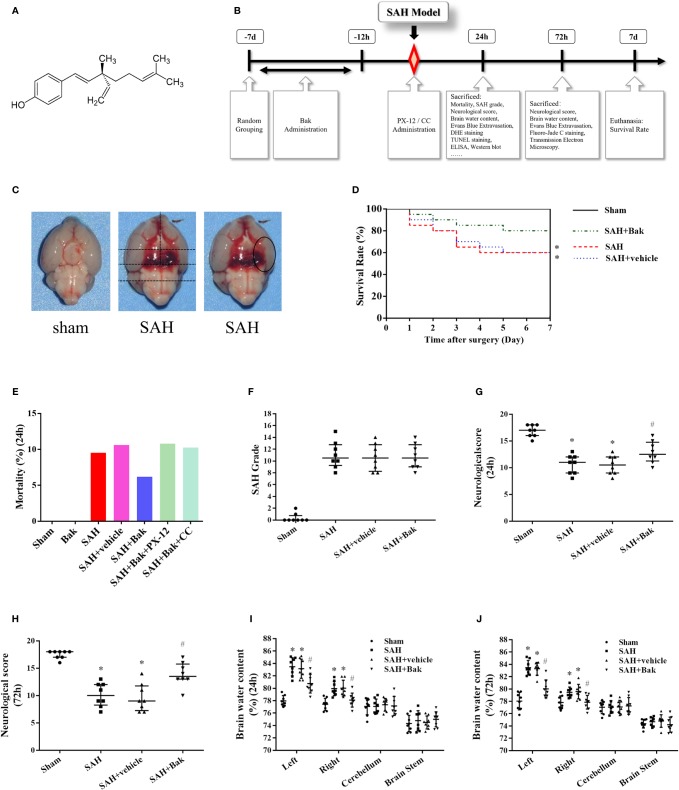
Experimental protocol and effect of Bak on mortality, neurological score, and brain water content in each group. **(A)** The chemical structure of Bak. **(B)** Experimental protocol. **(C)** The brain after SAH or sham. Blood clots can be seen in the ventral brain after SAH. The method to evaluate the SAH grading scores and the area observed after staining were showed. **(D)** Effect of Bak on the 7-day survival rate after SAH. Survival percentages in each day after injury are shown. Values are expressed as survival percentage. n = 20 for each group. **(E)** The mortality in each group. **(F)** SAH grading scores in each group. n = 8 for each group. **(G, H)** The neurological scores at 24 and 72 h after SAH. n=8 for each group. **(I, J)** Brain water content at 24 and 72 h after SAH. The brains are divided into four parts: the left hemisphere, the right hemisphere, the cerebellum, and the brain stem. The water content of each part is shown separately. n=8 for each group. Values of SAH grading score and neurological score are expressed as median and 25th–75th percentiles. Other values are expressed as mean ± SD. *P < 0.05 vs. sham group, ^#^*P < 0.05 vs*. SAH + vehicle group. Bak, bakuchiol; SAH, subarachnoid hemorrhage.

Bakuchiol (Bak), [(1E,3S)-3-ethenyl-3,7-dimethyl-1,6-oct adien-1-yl] phenol, an analog of resveratrol, is a prenylated phenolic monoterpene isolated from the seeds of *Psoralea corylifolia* L. (Leguminosae) (Feng et al., 2016; Xin et al., 2019) **Figure 1A**. It was initially identified in *Otholobium pubescens*, a kind of Peruvian medicinal plant used for the treatment of diabetes (Krenisky et al., 1999). Recently, Bak has been demonstrated to have numerous pharmacological properties, including the antioxidative and anti-inflammatory, antidiabetic, antiaging, and anticancer properties (Choi et al., 2010; Seo et al., 2013; Chaudhuri and Bojanowski, 2014; Li L. et al., 2017; Lim et al., 2019; Xin et al., 2019). For example, Bak maintains the activities of mitochondrial respiratory enzyme and protects the functions of mitochondrial against oxidative stress injury (Haraguchi et al., 2000). Bak treatment could also alleviate the edema, inflammation, and oxidative stress in the sepsis-induced acute lung injury (Zhang et al., 2017). Besides, Bak treatment could attenuate myocardial ischemia-reperfusion injury by attenuating mitochondrial oxidative damage and apoptosis *via* the activation of the SIRT1/PGC-1α signaling pathway (Feng et al., 2016). This strong antioxidative effect might be mediated by the terpenoid chain in its structure *via* a radical scavenging way (Adhikari et al., 2003). However, the effects of Bak on SAH remain unclear.

The present study aims to investigate the effects of Bak on EBI after SAH. The protective effects of Bak on BBB integrity, oxidative stress, cellular apoptosis, and neurological function during EBI were explored in an endovascular perforation SAH model in C57BL/6J mice. The roles of thioredoxin (Trx)/thioredoxin-interacting protein (TXNIP) and AMP-activated protein kinase (AMPK), which are crucial for the regulation of molecules in intracellular oxidative stress, were then studied by using their selective inhibitor, PX-12 and compound C (CC).

## Materials and Methods

### Animals and Ethics

Healthy adult male C57BL/6J mice weighing 20–25 g were obtained from the Animal Center of the Fourth Military Medical University. The mice were maintained on a 12 h light/dark cycle at approximately 22°C under pathogen-free conditions with given free access to food and water. All experiments were performed according to *The Guide for the Care and Use of Laboratory Animals* published by the US National Institutes of Health (National Institutes of Health Publication, No. 85–23, revised 1996) and had been approved by the Ethics Committee of the Fourth Military Medical University (NO. TDLL2017-04-192).

### Reagents

Bak, dihydroethidium (DHE), and 4′,6-diamino-2-phenylindole (DAPI) were purchased from Sigma-Aldrich (St. Louis, MO, USA). 1-Methylpropyl 2-imidazolyl disulfide (PX-12) was purchased from Selleck Chem (Houston, TX, USA). CC (ab146597) and rabbit polyclonal antibodies against gp91^phox^ (ab80508), 4-hydroxynonenal (4-HNE) (ab46545), cleaved caspase-3 (ab2302), claudin-5 (ab15106), occludin (ab216327), and zonula occludens-1 (ZO-1) (ab96587) were purchased from Abcam (Cambridge, UK). Rabbit monoclonal antibodies against B-cell lymphoma-2 (Bcl-2) (2870), Bcl-2-associated X protein (Bax) (14796), matrix metalloproteinase-9 (MMP-9) (13667S), Trx-1 (2429S), TXNIP (14715S), AMPK (2532S), and phospho-AMPK (Thr172) (D4D6D) were purchased from Cell Signaling Technology (Beverly, MA, USA). Rabbit polyclonal antibody against β-actin (AC006) was purchased from ABclonal Biotech (College Park, Maryland, USA). A terminal deoxynucleotidyl transferase uridine triphosphate (UTP) nick-end labeling (TUNEL) kit was purchased from Roche (Mannheim, Germany). Fluoro-Jade C (FJC) was purchased from Millipore (Temecula, USA). The enzyme-linked immune sorbent assay (ELISA) kits used to measure 8-hydroxy-2-deoxyguanosine (8-OHdG) and 3-nitrotyrosine (3-NT) levels were purchased from Cell Biolabs (San Diego, CA, USA). The kits used to measure glutathione peroxidase (GSH-Px), superoxide dismutase (SOD), and malondialdehyde (MDA) levels were purchased from the Institute of Jiancheng Bioengineering (Nanjing, Jiangsu, China). Human resource planning (HRP)-conjugated goat anti-rabbit secondary antibody was purchased from Bioworld Co. (Shanghai, China).

### Experimental Designs

First, mice were randomly assigned to four groups: the sham, SAH, SAH + vehicle, and SAH + Bak groups (**Figure 1B**). Sham or SAH operation was conducted in the appropriate groups. Bak was diluted to a concentration of 10 mg/ml in normal saline with a 2% volume of ethanol. Drugs were administered orally by gavage at a dosage of 50 mg/kg/day for seven consecutive days before the injury. The mice in the vehicle group were given equal doses of 2% ethanol dissolved in normal saline. Second, PX-12 (25 mg/kg), a selective Trx inhibitor, and CC (2 mg/kg), a selective AMPK inhibitor, were administered to mice in the SAH + Bak + PX-12 and SAH + Bak + CC groups through tail vein injection immediately after SAH. The sham + Bak group was also introduced to observe the effects of Bak on normal animals. Tissues were collected 24 or 72 h after injury for the following experiments. All of the experiments and statistical analyses were conducted by researchers blinded to the grouping.

### Subarachnoid Hemorrhage Animal Model

An endovascular perforation SAH model was developed by a method that has been described previously (Sozen et al., 2009; Kooijman et al., 2014). Briefly, animals were anesthetized with a mixture of isoflurane in 30% oxygen and 70% nitrous oxide (3% induction, 1.5% maintenance, v/v). Then, the left common carotid artery bifurcation was exposed, and the left external carotid artery was ligated and dissected. A nylon suture was inserted into the left internal carotid artery through the left external carotid artery stump and the left common carotid artery bifurcation. Resistance was encountered when the suture was located near the anterior communicating artery (ACA). The suture was then advanced approximately 3 more millimeters (total depth of 13–14 mm) to perforate the ACA. The suture was maintained in this position for 30 s before its withdrawal. A similar operation was performed on mice in the sham group, except that the suture was pierced less than 8 mm to avoid perforation of the ACA. The temperature was continuously monitored during the surgery. The animals were kept warm using a heating plate to maintain body temperature at 36.5–37.5°C during surgery and within 2 h after surgery. Meloxicam was given as analgesic after surgery, and 5% glucose dissolved in normal saline was given as nutritional support. To ensure the uniformity of damage, the mice with an SAH grading score ≤ 7 were excluded.

### Survival Rate and Subarachnoid Hemorrhage Grade

Twenty mice in each group were used to evaluate the postinjury survival rate. These animals were observed for 7 days after sham or SAH operation under pathogen-free conditions with free access to food and water. They were then euthanized after 7 days.

SAH grading scores were evaluated when the animals were sacrificed using a previously reported method (Sugawara et al., 2008). Briefly, the basal cisterns of the animals were divided into six segments (**Figure 1C**) and the Willis circle and basilar arteries were observed. Subarachnoid blood clotting and the arteries on each segment were evaluated and scored from 0 to 3 score (grade 0: no subarachnoid blood; grade 1: minimal subarachnoid blood; grade 2: moderate blood clot with recognizable arteries; grade 3: blood clot obliterating all arteries within the segment). The SAH grading score was the sum of the scores of each segment.

### Neurological Score

Neurological function was assessed using a modified Garcia’s neurological scoring system (Garcia et al., 1995; Zhao et al., 2017) at 24 or 72 h after surgery. In brief, the evaluation consisted of six tests of the following: spontaneous activity (0–3), symmetry in the movement of all four limbs (0–3), symmetry in the movement of forelimbs (0–3), climbing (0–3), response to trunk stimulation (0–3), and response to whisker stimulation (0–3). The maximum score was 18; the minimum score was 0. Higher scores indicated better function.

### Brain Water Content

The brain water content was tested by a previously described method (Xi et al., 2001; Jin et al., 2017) using the following formula: the brain water content (%) = [(wet weight − dry weight)/wet weight] ×100%. The mice were deeply anesthetized and sacrificed 24 or 72 h after surgery. Their brains were removed 24 or 72 h after injury and divided into four parts: the left hemisphere, the right hemisphere, the cerebellum, and the brain stem. Then, the water contents of each part were tested separately. Tissues were weighed immediately to obtain the wet weight, followed by drying at 95 to 100°C for 72 h and then weighing to obtain the dry weight.

### Evans Blue Extravasation

Evans blue (EB) extravasations were tested 24 and 72 h after injury using a spectrophotometer (λ = 610 nm) to evaluate the permeability of the BBB. Briefly, 4 ml/kg of 2% (w/v) EB dye was injected into the right tail vein 3 h before the mice were sacrificed. Then, the animals were perfused transcardially with 50 ml of ice-cold 0.1 M phosphate-buffered saline (PBS, pH 7.4) under anesthesia to remove intravascular EB dye. The ipsilateral cortex was removed and homogenized in PBS and an equal volume of trichloroacetic acid to precipitate the protein. After 5 min, the samples were centrifuged, and the supernatants were extracted and used to measure the absorbance (Zhang et al., 2014; Zhang et al., 2015a).

### Dihydroethidium Staining

DHE staining was conducted to detect the superoxide anion, which reflected the oxidant stress levels in the tissue. Animals were perfused transcardially with PBS under anesthesia 24 h after SAH. Samples were immediately frozen at −80°C and sliced into 15 μm thick coronal brain slice with a freezing microtome (CM 1950, Leica, German). With reference to *The Mouse Brain in Stereotaxic Coordinates (Second Edition) (ACADEMIC PRESS)*, the slices at 0.58–2.5 mm posterior to bregma were selected. The slices were dyed with DHE for 30 min. Then, the ventral side of the left hemisphere was observed with a laser scanning confocal microscope (A1 Si, Nikon, Japan) (**Figure 1C**). The representative images were obtained from the slices located at about 2 mm posterior to bregma.

### Assay of Malondialdehyde Content and the Superoxide Dismutase and Glutathione Peroxidase Activities

The brains were removed after their perfusion with PBS 24 h after SAH, and the ipsilateral cortex was homogenized to detect the levels of MDA and the activities of oxidative stress-related enzymes (SOD and GSH-Px). According to the instructions of commercial kits, the MDA levels were tested by the reaction of MDA with thiobarbituric acid under acidic conditions and a high temperature, following which the absorbance was detected. SOD activity was tested by WST-1 method following the instruction of commercial kits. The GSH-Px activity was tested by detecting the reduction of NADPH in the reaction system following the instructions of a commercial kit.

### Concentrations of 3-Nitrotyrosine and 8-Hydroxy-2-Deoxyguanosine

ELISA kits were used to evaluate the levels of 3-NT and 8-OHdG in injured tissues at 24 h after SAH. Briefly, samples or standards were incubated with primary antibody at 4°C overnight, secondary antibody at room temperature for 1 h, and a substrate solution at the room temperature for 15 min in the dark. Finally, a solution to terminate the reaction was added to each sample. A SpectraMax M2 spectrometer (Molecular Devices, Sunnyvale, CA, USA) was used to measure the absorbance and calculate the protein level.

### Transmission Electron Microscopic Observation

Samples were prepared following previously reported methods (Li X. et al., 2017). Briefly, 72 h after SAH induction, the mice were anesthetized and perfused with 50 ml of ice-cold PBS and 60 ml of ice-cold 4% paraformaldehyde (PFA). Then, the brains were removed. The injured cortical tissues were cut perpendicular to the long axis and trimmed into 1.5mm×1.5mm×3mm blocks. Then, the specimens were fixed for 12 h in 4% glutaraldehyde, postfixed for 1 h in 1% osmium tetroxide, dehydrated through graded ethanol, and embedded in resin. Specimens were cut into 80 nm sections by an ultramicrotome (Leica, Vienna, Austria). The ultrathin sections were fixed on 200 slot grids coated with Pioloform membranes and observed with a JEM-1400 electron microscope (JEOL, Tokyo, Japan). Micrographs were captured with a charge-coupled device camera (Olympus, Tokyo, Japan).

### Terminal Deoxynucleotidyl Transferase Uridine Triphosphate Nick-End Labeling Assay

A TUNEL kit was used to detect cell apoptosis in the injured cortex. The animals were perfused with PBS and PFA 24 h after the injury as described above. Their brains were cautiously removed, fixed in 4% PFA for 12 h and dehydrated in sucrose solutions at different concentrations (10, 20, and 30%). The tissues were sliced into 25 μm thick slices with a freezing microtome. The slices were incubated with 0.3% hydrogen peroxide for 30 min at room temperature, 0.25% pancreatin for 45 min at 37°C, TUNEL reaction solution for 60 min at 37°C and DAPI (5 μg/ml) staining solution for 10 min at 37°C in a humidified box in the dark. With reference to *The Mouse Brain in Stereotaxic Coordinates (Second Edition) (ACADEMIC PRESS)*, the slices at 0.58–2.5 mm posterior to bregma were selected. The ventral side of the left hemisphere was observed with a confocal microscope (**Figure 1C**). The representative images were obtained from the slices located at about 2 mm posterior to bregma. The apoptotic index was reflected by the ratio of TUNEL-positive cells to DAPI-positive cells.

### Fluoro-Jade C Staining

FJC staining was performed to detect neuronal degeneration in the tissues 72 h after injury (Bai et al., 2018). Briefly, the tissues were perfused, collected, fixed, dehydrated, and sliced as described above. The observed slices and areas were selected according to the method described above. Selected sections were incubated with 1% NaOH in 80% ethanol for 5 min and then rehydrated with 70% ethanol for 2 min and distilled water for 2 min. The slices were then incubated with 0.06% KMnO_4_ for 10 min, rinsed with distilled water for 3 min, and incubated with a 0.0001% FJC solution for 15 min. Finally, the slices were washed three times with distilled water for 1 min each. The ventral side of the left hemisphere was observed and images were obtained using a confocal microscope (**Figure 1C**). FJC-positive neurons were counted and calculated.

### Western Blot Analysis

The ipsilateral cortical samples collected 24 h after injury were sonicated and homogenized in a mixture of lysis buffer and 1% protease inhibitor for 30 min and then centrifuged for 15 min at 12,000 rpm. Equal amounts of protein (25 µg) were separated on 8–15% sodium dodecyl sulfate (SDS)-polyacrylamide gels and transferred onto polyvinylidene difluoride (PVDF) membranes (Millipore Corporation, USA). The PVDF membranes were blocked in 5% nonfat dry milk/TBST (Tris-buffered saline, 0.1% Tween 20) for 90 min at room temperature and then incubated with rabbit anti-claudin-5 (1:1,000), anti-occludin (1:1,000), anti-ZO-1 (1:1,000), anti-MMP-9 (1:1,000), anti-gp91phox (1:500), anti-4-HNE (1:500), anti-Bcl-2 (1:1,000), anti-Bax (1:1,000), anti-cleaved caspase-3 (1:1,000), anti-Trx 1 (1:1,000), anti-TXNIP (1:1,000), anti-AMPK (1:1,000), anti-phospho-AMPK (1:1,000), and anti-β-actin (1:1,000) primary antibodies overnight at 4℃, followed by HRP-conjugated goat anti-rabbit (1:5,000) secondary antibody for 90 min at room temperature. Finally, the membranes were detected with the Bio-Rad imaging system (Bio-Rad, Hercules, CA, USA).

### Statistical Analysis

GraphPad Prism 6 (GraphPad Software, San Diego, CA, USA) and SPSS 18.0 (SPSS, Chicago, IL, USA) was used for analysis. The survival rate was analyzed with the log-rank (Mantel-Cox) test. SAH grading scores and neurological scores are expressed as medians and 25th–75th percentiles and were analyzed by the Kruskal–Wallis one-way analysis of variance (ANOVA) on ranks, followed by Tukey’s *post hoc* analysis. Means ± SDs are provided to describe other data. One-way ANOVA, followed by Tukey’s *post hoc* analysis, and Bonferroni multiple comparison tests were used for intergroup comparisons. Multiple group comparisons were tested by one-way ANOVA followed by Tukey’s honest significant difference (HSD) *post hoc* test. Differences for which *P < 0.05* were considered statistically significant.

## Results

### Bak Increases the Survival Rate and Alleviates Neurological Functional Deficits and Brain Edema After Subarachnoid Hemorrhage

The numbers of animals in each group used in the present study were described in **Table 1**. The overall mortality rate within 24 h after surgery in the sham, Bak, SAH, SAH + vehicle, SAH + Bak, SAH + Bak + PX-12, and SAH + Bak + CC groups were 0% (0/92), 0% (0/6), 9.5% (10/105), 10.6% (12/113), 6.2% (7/113), 10.8% (4/37), and 10.3% (4/39), respectively (**Figure 1E**). No animal was excluded from the experiment to observe the 7-day survival rate. The survived animals with insufficient brain injury were excluded from other experiments. Finally, 10, 9, 14, 3, and 4 animals were excluded in the SAH, SAH + vehicle, SAH + Bak, SAH + Bak + PX-12, and SAH + Bak + CC groups, respectively; 9, 12, 7, 4, 5 animals died before being sacrificed in the SAH, SAH + vehicle, SAH + Bak, SAH + Bak + PX-12, and SAH + Bak + CC groups, respectively (**Table 1**).

**Table 1:** The numbers of animals in each group.

	sham	sham+Bak	SAH	SAH			
				Vehicle	Bak	Bak+PX-12	Bak+CC
**Included**							
**Survival rate**	20	0	20	20	20	0	0
**Brain water content**	16	0	16	16	16	8	8
**Evans blue extravasation**	14	0	14	14	14	7	7
**DHE staining**	6	0	6	6	6	0	0
**Oxidative stress marker detection**	6	0	6	6	6	6	6
**Western Blot**	12	6	6	12	12	6	6
**TUNEL**	6	0	6	6	6	0	0
**FJC staining**	6	0	6	6	6	0	0
**Transmission electron microscopy**	6	0	6	6	6	3	3
**In total**	92	6	86	92	92	30	30
**Died before being sacrificed**	0	0	9	12	7	4	5
Excluded*	0	0	10	9	14	3	4

*No animal was excluded from the experiment to observe the survival rate. The animal with a SAH grading score ≤ 7 were excluded in other experiments. Bak, bakuchiol; SAH, subarachnoid hemorrhage; PX-12, 1-methylpropyl 2-imidazolyl disulfide; CC, compound C; DHE, dihydroethidium; TUNEL, terminal deoxynucleotidyl transferase UTP nick-end labeling; FJC, Fluoro-Jade C.

The 7-day survival rates in the sham, SAH, SAH + vehicle, and SAH + Bak groups were 100, 60, 60, and 80%, respectively (**Figure 1D**). Blood clots were clearly visible around the Willis circle and ventral brainstem of animals in the SAH, SAH + vehicle, and SAH + Bak groups. There was no significant difference in SAH grading scores between the SAH, SAH + vehicle, and SAH + Bak groups (**Figure 1F**).

Functional deficits, which were evaluated by neurological scores, and the degree of brain edema, which was evaluated by determining the brain water content, were measured 24 and 72 h after injury. SAH caused apparent neurological deficits and brain edema. There were no significant differences in neurological scores or brain water content between the SAH and SAH + vehicle groups. Bak significantly improved neurological deficits and decreased the brain water content at 24 and 72 h after SAH (**Figures 1G–J**). The data of 24 h—brain water content/neurological scores and 72 h—brain water content/neurological scores were collected from different batches of animals. Thus, the difference between the injuries at 24 and 72 h was not compared.

### Bak Protects Blood-Brain Barrier Integrity After Subarachnoid Hemorrhage

**Figure 2:**
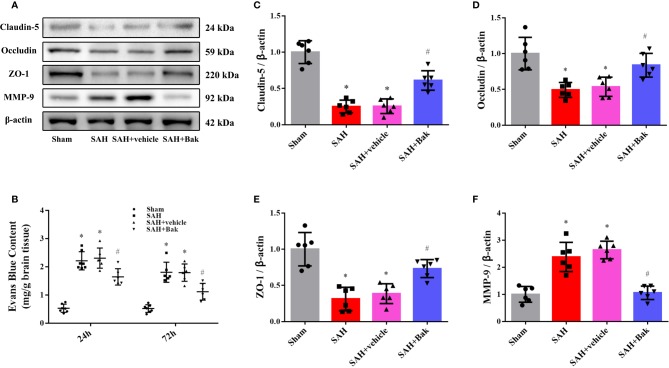
The effect of Bak on BBB integrity after SAH. **(A)** Representative western blot images of the level of claudin-5, occludin, ZO-1, and MMP-9 at 24 h after SAH. n=6 for each group. **(B)** The EB extravasations at 24 and 72 h after injury. n=7 for each group. **(C–F)** Statistical analysis of the protein level of claudin-5, occludin, ZO-1, and MMP-9. Values are expressed as mean ± SD. ^*^P < 0.05 vs. sham group, ^#^*P < 0.05 vs*. SAH + vehicle group. Bak, bakuchiol; SAH, subarachnoid hemorrhage; BBB, blood-brain barrier; ZO-1, zonula occludens-1; MMP-9, matrix metalloproteinase-9; EB, Evans blue.

EB extravasation was tested 24 and 72 h after injury to evaluate the permeability of BBB. A sharp increase in EB extravasation was observed in the SAH and SAH + vehicle groups (*vs.* the sham group, *P* < 0.05), which was alleviated by Bak (**Figure 2B**). In addition, decreases in levels of the tight junction proteins claudin-5, occludin, and ZO-1 were observed in the SAH and SAH + vehicle groups (*vs.* the sham group, *P* < 0.05) 24 h after injury, which were offset by Bak. The MMP-9 level was increased in the SAH group (*vs.* the sham group, *P* < 0.05), but significantly reduced by Bak (**Figures 2A, C–F**).

### Bak Ameliorates Oxidative Stress After Subarachnoid Hemorrhage

**Figure 3:**
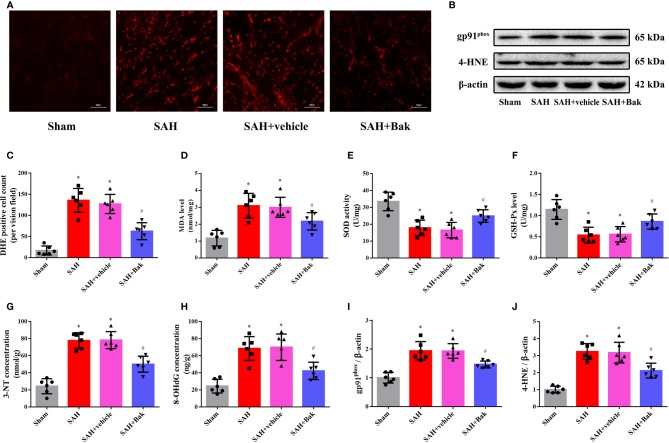
The effect of Bak on the oxidative stress 24 h after SAH. **(A, C)** Representative images and quantitative analyses of DHE staining. Scale bar = 50 μm. **(D–F)** The effect of Bak on the level of MDA, and on the activity of SOD and GSH-Px after SAH. **(G, H)** The effect of Bak on 8-OHdG and 3-NT concentrations after SAH. **(B, I, J)** The effects of Bak on the gp91^phox^ and 4-hydroxynonenal (4-HNE) after SAH. The representative western blot images and statistical analysis of the protein levels of gp91^phox^ and 4-HNE are shown. Values are expressed as mean ± SD, n=6 for each group. ^*^P < 0.05 *vs.* sham group, ^#^*P < 0.05 vs*. SAH + vehicle group. Bak, bakuchiol; SAH, subarachnoid hemorrhage; DHE, dihydroethidium; MDA, malondialdehyde; SOD, superoxide dismutase; GSH-Px, glutathione peroxidase; 8-OHdG, 8-hydroxy-2-deoxyguanosine; 3-NT, 3-nitrotyrosine.

The proportion of DHE-positive cells was dramatically increased after SAH and significantly decreased by Bak (**Figures 3A, C**). The levels of MDA were significantly increased after SAH but significantly decreased following Bak administration (**Figure 3D**). In addition, the SOD and GSH-Px activities were impaired by SAH and remarkably enhanced by Bak administration (**Figures 3E, F**). Moreover, the levels of 3-NT and 8-OHdG were also increased after SAH and decreased significantly by Bak administration (**Figures 3G, H**). The protein levels of gp91^phox^ and 4-HNE in the injured cortices were also increased 24 h after SAH and reduced by Bak treatment (**Figures 3B, I, J**).

### Bak Attenuates Neuronal Damage After Subarachnoid Hemorrhage

**Figure 4:**
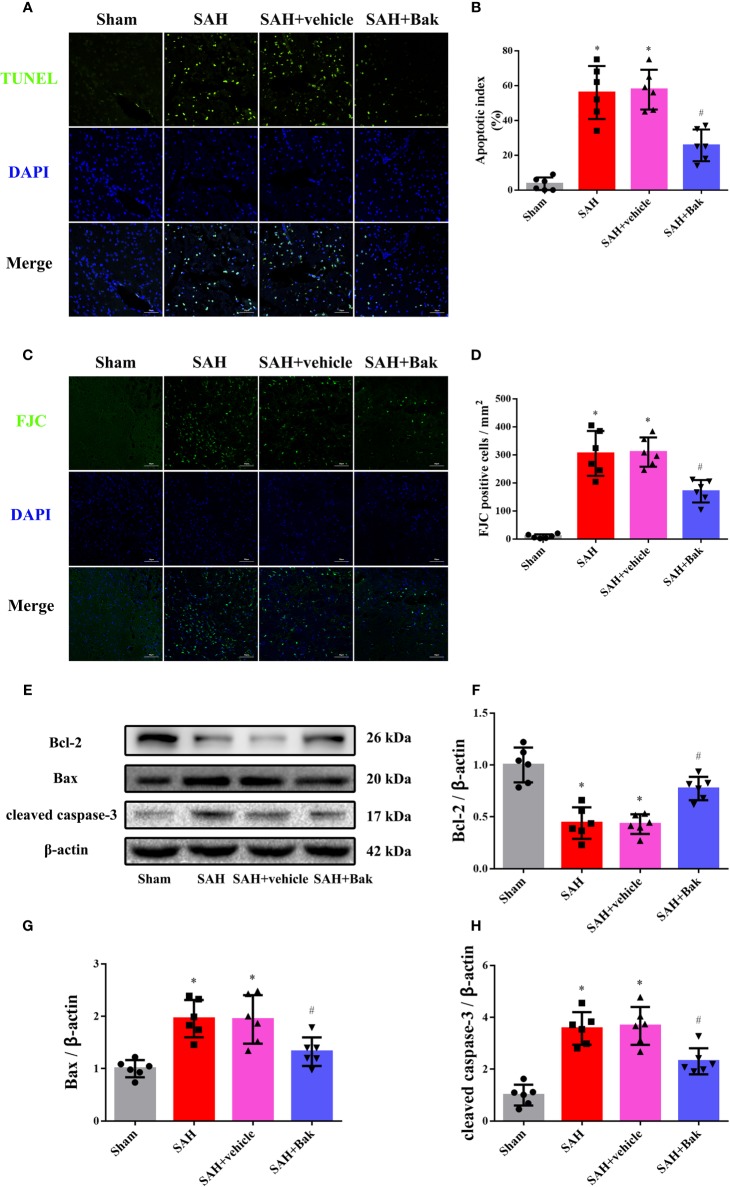
The effects of Bak on cellular apoptosis and neuronal degeneration following SAH. **(A, B)** Representative images of TUNEL staining and quantitative analyses of TUNEL positive cells. Scale bar = 50 μm. **(C, D)** Representative images of FJC staining and quantitative analyses of FJC positive cells. Scale bar = 50 μm. **(E–H)** The effects of Bak on the apoptosis signaling 24 h after SAH. The representative western blot images and statistical analysis of the protein levels of Bcl-2, Bax, and cleaved caspase-3 are shown. Values are expressed as mean ± SD, n=6 for each group. ^*^P < 0.05 vs. sham group, ^#^*P < 0.05 vs*. SAH + vehicle group. Bak, bakuchiol; SAH, subarachnoid hemorrhage; TUNEL, terminal deoxynucleotidyl transferase UTP nick-end labeling; FJC, Fluoro-Jade C.

TUNEL staining was performed to observe cellular apoptosis. The proportion of apoptotic cells was significantly increased after SAH. Bak significantly decreased the apoptotic index (**Figures 4A, B**). Previously, it was believed that the apoptosis mainly occurred on neurons after stroke. However, the latest research showed that the apoptosis and cell loss also occurred on glial cells (Chen et al., 2017; Ma et al., 2017; Sekerdag et al., 2018). Thus the FJC staining was further performed to quantify the degenerated neurons. The number of FJC-positive degenerated cells was significantly increased after SAH. This degeneration was alleviated by Bak administration (**Figures 4C, D**).

The protein levels of Bax, Bcl-2, and cleaved caspase-3 in injured tissues were tested by western blotting. The protein levels of Bax and cleaved caspase-3 were increased 24 h after SAH, which were partially offset by Bak. However, the protein level of Bcl-2 was decreased 24 h after SAH, which was significantly ameliorated by Bak (**Figures 4E–H**).

### The Role of Trx/TXNIP and AMPK in the Protective Effects of Bak Against Subarachnoid Hemorrhage

**Figure 5:**
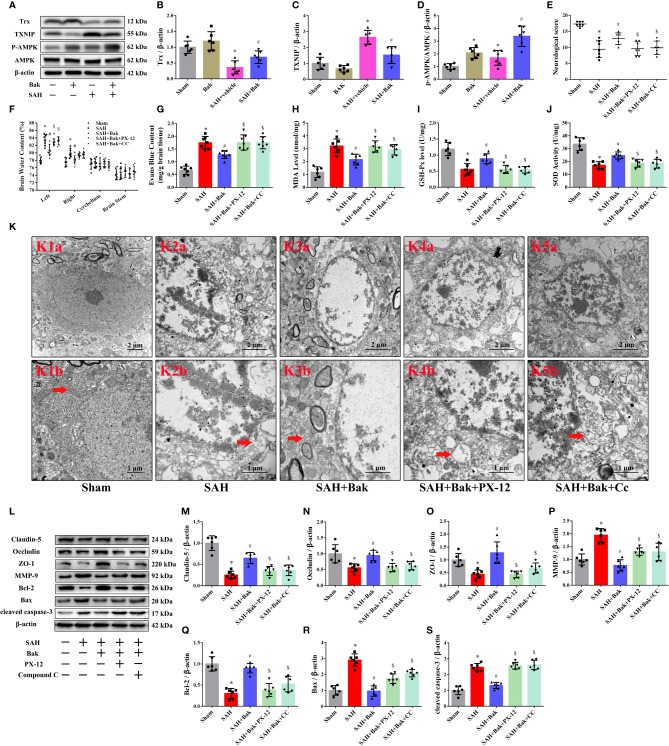
The role of Trx/TXNIP and AMPK in the protective effect of Bak after SAH. **(A–D)** The representative western blot images and statistical analysis of the protein levels of Trx and TXNIP and the phosphorylation of AMPK at 24 h after SAH. n=6 for each group. The protein levels of Trx and the phosphorylation levels of AMPK are up-regulated significantly by Bak (*vs.* the SAH + vehicle group). Then, the PX-12 and CC were used to identify the role of Trx and AMPK in the protective mechanism of Bak. **(E)** The neurological scores at 24 h. n=7 for each group. Values are expressed as median and 25th–75th percentiles. **(F)** Brain water content at 24 h. n=8 for each group. **(G)** Evans blue (EB) extravasation at 24 h. n=7 for each group. **(H–J)** The level of MDA, and the activity of SOD and GSH-Px in each group. n=6 for each group. **(K)** The ultrastructure of neurons in each group. K1b–K4b are the enlargements representative ultrastructure of neurons in K1a–K4a respectively. Scale bar = 2 μm in K1a–K4a, 1 μm in K1b–K4b. Arrows indicate the mitochondria. **(L–S)** The representative western blot images and statistical analysis of the protein levels of claudin-5, occludin, ZO-1, MMP-9, Bcl-2, Bax, and cleaved caspase-3 at 24 h. n=6 for each group. Values are expressed as mean ± SD except the neurological score, *P < 0.05 vs. sham group, ^#^*P < 0.05 vs*. SAH + vehicle group, ^$^*P < 0.05 vs*. SAH + Bak group. Bak, bakuchiol; SAH, subarachnoid hemorrhage; Trx, thioredoxin; TXNIP, thioredoxin-interacting protein; AMPK, AMP-activated protein kinase; PX-12, 1-methylpropyl 2-imidazolyl disulfide; CC, compound C; MDA, malondialdehyde; SOD, superoxide dismutase; GSH-Px, glutathione peroxidase; ZO-1, zonula occludens-1; MMP-9, matrix metalloproteinase-9.

Western blotting was conducted 24 h after SAH to explore the role of Trx, TXNIP, and AMPK in the protective effects of Bak. The protein level of Trx was significantly decreased after SAH and increased after Bak administration. In contrast, the protein level of TXNIP was significantly increased after injury and decreased after Bak administration. In addition, the phosphorylation of AMPK was significantly increased after SAH. Bak administration further increased the level of phosphorylated AMPK (*vs.* the SAH + vehicle group, *P < 0.05*) Besides, an increase of AMPK phosphorylation also observed in the sham + Bak group (*vs.* the sham group, *P < 0.05*) (**Figures 5A–D**).

Then, PX-12, a Trx inhibitor, and CC, an AMPK inhibitor, were used as negative controls to further explore the roles of AMPK and Trx/TXNIP in this mechanism. The neurological score was decreased, and the brain water content and EB extravasation were obviously increased after the administration of PX-12 or CC compared to those in the SAH + Bak group (**Figures 5E–G**). The MDA level was significantly increased by PX-12 and CC (*vs.* the SAH + Bak group, *P < 0.05*). In contrast, the activities of GSH-Px and SOD were downregulated by PX-12 and CC (*vs.* the SAH + Bak group, *P < 0.05*) (**Figures 5H–J**). Meanwhile, transmission electron micrographs were used to observe ultrastructural changes in the mitochondria of neurons. Neurons in the SAH + Bak + PX-12 and SAH + Bak + CC groups were characterized by the loss of mitochondrial cristae, swollen mitochondria, and morphological changes in the endoplasmic reticulum, which was similar to neurons in the SAH group (**Figure 5K**).

BBB integrity and cellular apoptosis were also tested 24 h after injury. The increased protein levels of claudin-5, occludin, ZO-1, and Bcl-2 after Bak administration were significantly reduced by PX-12 or CC. In addition, the levels of MMP-9 and Bax were increased significantly by PX-12 or CC (**Figures 5L–S**).

## Discussion

SAH accounts for 5% of all strokes and occurs at a fairly young age (van Gijn et al., 2007). Patients with SAH often have cognitive impairments, which severely affect their ability to work and quality of life (Macdonald and Schweizer, 2017). However, the current SAH treatment strategy does not achieve a satisfactory functional outcome. Thus, the need to find better treatment is urgent.

EBI is the most important cause of disability and death after SAH. The treatment of EBI may successfully attenuate some of the devastating secondary injuries and improve the outcome of SAH patients^5^. Thus, attenuating EBI is the main goal of SAH treatment and a crucial method to reduce disability and mortality (Cahill and Zhang, 2009).

**Figure 6:**
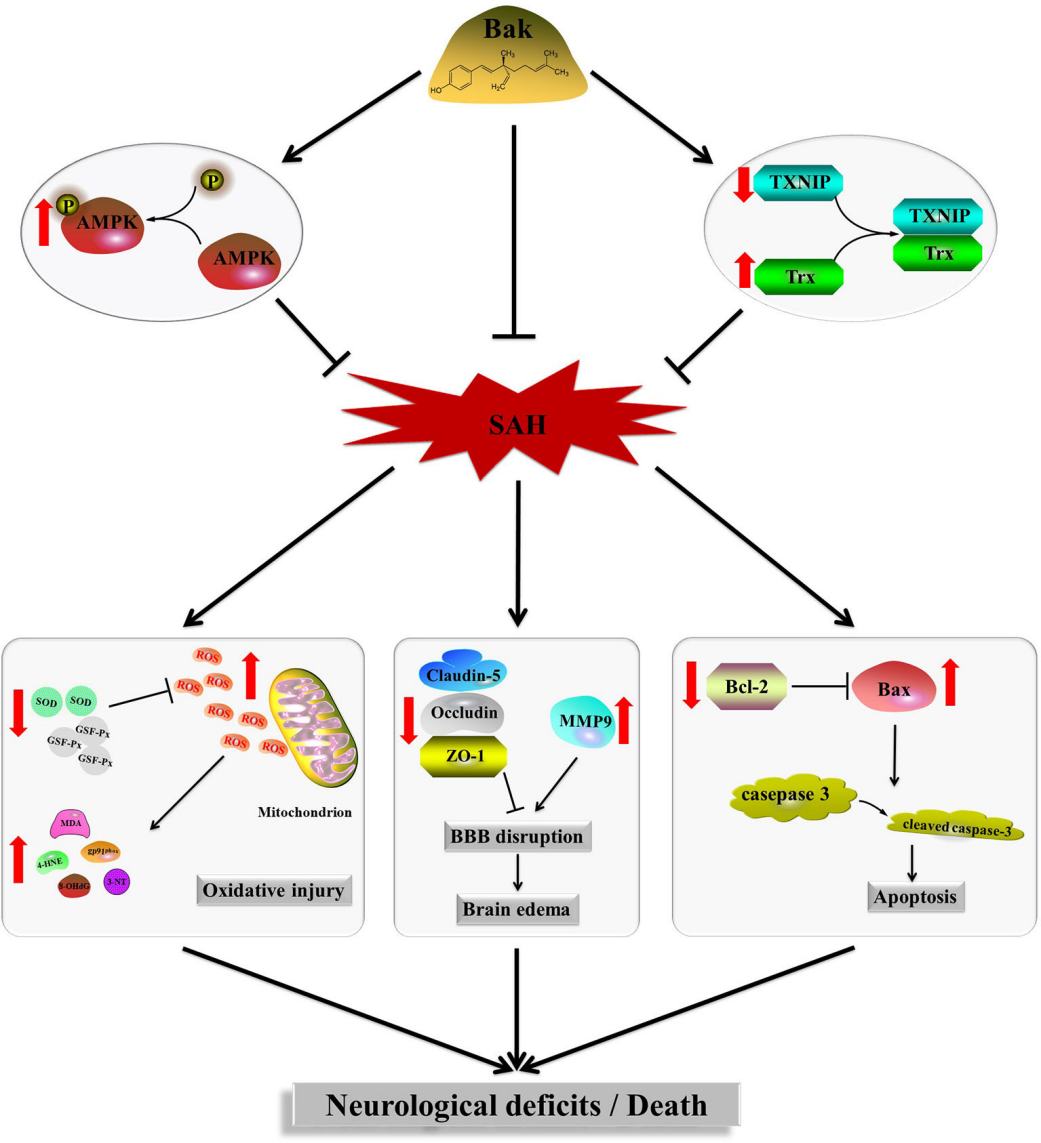
Signaling pathway of Bak’s neuroprotective effect against SAH injury which is suggested in the present study. Bak promotes the phosphorylation of AMPK, upregulates the protein level of Trx, and downregulated the protein level of TXNIP, thus alleviates the oxidative stress, cellular apoptosis, brain edema, and neurological deficits after SAH. Bak, bakuchiol; SAH, subarachnoid hemorrhage; Trx, thioredoxin; TXNIP, thioredoxin-interacting protein; AMPK, AMP-activated protein kinase.

Bak, a prenylated phenolic monoterpene, is used in both traditional Chinese medicine and traditional Indian medicine (Chen et al., 2010). Recently, Bak was demonstrated to have multiorgan protective effects through a variety of pharmacological activities (Xin et al., 2019). In the present study, oral administration of Bak 1) reduced the mortality rate and improved the neurological function of animals after SAH, 2) attenuated disruption to the BBB and brain edema caused by SAH, 3) reduced the superoxide production, alleviated oxidative stress, and protected the mitochondrial ultrastructure during EBI, 4) attenuated SAH-induced cellular apoptosis and neuron damage, and 5) regulated the protein levels of Trx and TXNIP and the phosphorylation of AMPK. Moreover, both PX-12, a selective Trx inhibitor (Ji et al., 2019), and CC, a selective AMPK inhibitor (Oliveira et al., 2012; Guo et al., 2018), reversed the protective effects of Bak. To summarize, the present study confirmed that Bak can inhibit oxidative stress, attenuate cellular apoptosis, and ameliorate BBB disruption *via* regulating the protein level of Trx and the activity of AMPK after SAH (**Figure 6**).

The pathological mechanisms of EBI include oxidative stress, inflammation, cellular apoptosis, disruption of the BBB, and microvascular dysfunction (Cahill et al., 2006; Sehba et al., 2012). Intercellular contacts between cerebral microvessel endothelial cells participate in the formation of the BBB and are critical to maintaining the brain’s structure and function (Keep et al., 2018). These intercellular contacts include tight junctions, the stability of which is maintained by complex networks of occludin, claudin-5, ZO-1 and junctional adhesion molecule (JAM), and adherens junctions, which consist of vascular endothelial (VE) cadherins (Altay et al., 2012; Keep et al., 2018). Proteins in the MMP family are involved in the breakdown of extracellular matrix in the pathological processes of intracerebral hemorrhage (Zhao et al., 2006; Cao et al., 2016). Disruption of the BBB induces vasogenic edema, allows leukocyte extravasation, and allows neurotoxic and vasoactive compounds to leak into the brain (Keep et al., 2018). The BBB disruption and brain edema in EBI after SAH were alleviated by Bak in the present study.

Additionally, the excessive production and release of free radicals, with a weakened scavenger system, disrupt the BBB, leading to brain edema (Park et al., 2004). Reactive oxygen species (ROS) are chemically reactive chemical species containing oxygen including peroxides, superoxide, hydroxyl radical, singlet oxygen, and alpha-oxygen (Hayyan et al., 2016). It is produced in mitochondria by the electron transport chain under physiological conditions. The overload of ROS not only induces oxidative stress damage but also mediates inflammation and apoptosis (Bolanos et al., 2009; Forrester et al., 2018). EBI induces oxidative damage through inhibiting intrinsic antioxidant systems and increasing the production of ROS (Zhang et al., 2016; Liu H. et al., 2017). The production of ROS leads to serious tissue damage by promoting lipid peroxidation, DNA damage, and protein modification (Liu H. et al., 2017). The superoxide anion, as a representative of ROS, was detected by DHE staining to reflect the oxidant stress levels in the tissue in the present study.

Lipid peroxidation is a consequence of free radical-mediated injury in the brain. MDA, 3-NT, and 4-HNE are products of the lipid peroxidation chain reaction and markers which reflect the degree of tissue lipid peroxidation (Gutteridge and Halliwell, 1990; Ayala et al., 2014). 8-OHdG is widely used as a sensitive marker of DNA damage (Di Minno et al., 2016). gp91^phox^, a member of the NADPH oxidase (NOX) family, is the primary catalytic subunit of NADPH oxidase which produces reactive oxygen species (Hasegawa et al., 2017). SOD is a member of the enzymatic antioxidative pathway by which the dismutation of superoxide anions into hydrogen peroxide (H_2_O_2_) and oxygen (O_2_) is catalyzed. GSH-Px also has notable antioxidative stress effects. Under physiological conditions, SOD, GSH-Px, and other antioxidant molecules comprise the intrinsic antioxidant system and fight against harmful reactive oxygen species (Wu et al., 2004; Poprac et al., 2017). The protective effects of Bak are mainly derived from its strong antioxidative properties (Adhikari et al., 2003; Xin et al., 2019). In the present study, Bak simultaneously increased the activities of SOD and GSH-Px and decreased the levels of superoxide production, MDA, 3-NT, 8-OHdG, gp91^phox^, and 4-HNE in EBI.

ROS can be produced in the endoplasmic reticulum, mitochondria, cytoplasm, and peroxisome. The NOX family is one of the important sources of cytoplasmic ROS, a cornerstone of cellular signaling. Mitochondrial ROS are a natural byproduct of electron transfer in the respiratory chain. Proper respiratory chain function in mitochondria requires a delicate balance between the prooxidant and antioxidant systems (Forrester et al., 2018). In our study, the morphology of mitochondria and endoplasmic reticulum in neurons was observed. Mitochondria were found to be badly impaired, characterized by the loss of mitochondrial cristae, mitochondrial swelling, and collapsed cristae, during EBI, which were notably reversed by Bak administration. The normal morphology of the endoplasmic reticulum was also observed after Bak treatment.

The Trx/TXNIP system has been suggested to be an important contributor to enzymatic systems in ROS generation and oxidative stress (Ji et al., 2019). Trx2, the main ROS-scavenging enzyme in mitochondria, balances ROS levels and maintains mitochondrial function. Trx1, which is located mainly in the cytoplasm, is usually induced as a response to oxidative stresses (Yoshihara et al., 2014). TXNIP, an endogenous inhibitor of the Trx system, directly binds to Trx1/Trx2 and inhibits their activity through disulfide exchange (Yoshihara et al., 2014; Nasoohi et al., 2018). The inhibition or the deletion of TXNIP is found to be neuroprotective in cerebrovascular and neurodegenerative diseases (Nasoohi et al., 2018). In the present study, the Trx/TXNIP system was involved in the antioxidative mechanism of Bak during EBI.

Cytoplasmic ROS also regulate the activity of AMPK (Hinchy et al., 2018), a central regulator of metabolic functions including lipid metabolism and mitochondrial function (Forrester et al., 2018; Herzig and Shaw, 2018). AMPK is activated by its phosphorylation at threonine-172 and plays a neuroprotective role after SAH (Lin and Hardie, 2018; Enkhjargal et al., 2019). In the present study, an increase in AMPK phosphorylation was observed after Bak administration. Besides, the upregulation of AMPK phosphorylation occurs after SAH in the present study, which has also been reported previously (Osuka et al., 2009; Xu et al., 2019; Xu et al., 2020). In our opinion, this phenomenon may be caused by the compensation mechanism during which the self-protecting signal pathways have been activated to tolerate the stress. In the present study, this mechanism was further enhanced by Bak. More importantly, the protective effects of Bak against BBB disruption, mitochondrial impairment, oxidative stress, and cellular apoptosis were reversed by CC and PX-12.

Cellular apoptosis is the result of oxidative stress-associated lipid peroxidation, protein breakdown, and DNA damage in neurons and endothelial cells during EBI, which leads to neurological deficits (Yuksel et al., 2012; Zhang et al., 2015b). The Bcl-2 family proteins mediate the mitochondrial apoptotic pathway by regulating the release of cytochrome C. The overexpression of Bcl-2 protects cells from apoptosis, whereas the upregulation of Bax reduces the protective effects of Bcl-2 and promotes cell death (Li et al., 2016). We evaluated the expressions of Bcl-2 and Bax and the activity of caspase-3 after SAH. In the present study, as shown by TUNEL staining and western blotting, Bak protected against apoptosis, which strongly contributed to the alleviation of neurological deficits after SAH.

Previously, it was believed that the apoptosis mainly occurred on neurons after stroke. However, the latest research showed that the apoptosis and cell loss also occurred on glial cells (Chen et al., 2017; Ma et al., 2017; Ning et al., 2017; Sekerdag et al., 2018). Strictly, TUNEL staining alone could not reflect the entire destruction of neurons. However, the FJC staining, detecting the overall neurodegeneration, could reflect the degree of neuron damage more comprehensively. It has been reported that the distribution and degree of FJC-positive cells after SAH were remarkably larger than TUNEL-positive cells (Xie et al., 2015).

The oxidative stress and BBB disruption begin within a few minutes to several hours after stroke and peaks quickly during EBI. By contrast, an important feature of SAH is that there is a delayed phase of brain injury at 3–14 days after hemorrhage in about a third of patients (Macdonald and Schweizer, 2017). The neuronal destruction continues from the EBI phase to the delayed injury phase, resulting in the continuous neurological deterioration (Coulibaly and Provencio, 2020). Considering that neurons are hard to regenerate, this persistent neuronal irreversible structural damage and loss of neurological functions worsen the SAH patients’ poor neurological functional outcomes. Therefore, the FJC staining and electron microscopy were conducted at 72 h after SAH to further evaluate the degree of neuron damage in the present study. Whereas, the long-term changes after 3 days in neuronal structure and neurological functions were not observed, which is a limitation.

With numerous pharmacological properties, Bak exerts a multiorgan protective effect to alleviate damage to the brain (Xin et al., 2019), liver, cardiovascular system, skin, and retina (Kim et al., 2013; Kim S. et al., 2016; Xin et al., 2019). Previous studies have demonstrated that Bak could play anti-neuroinflammatory effects on activated microglia by the inhibition of the p38 mitogen-activated protein kinases (MAPK)/extracellular signal-regulated kinase 1/2 (ERK) pathways (Lim et al., 2019). ERK could inhibit the activity of AMPK[18]. This evidence is consistent with our findings. Moreover, Bak is proved to be able to suppress lipopolysaccharide (LPS)-stimulated nitric oxide production in LPS-treated BV-2 microglia and shows potent inhibitory activity against hydrogen peroxide-induced cell death in HT22 hippocampal cells (Kim Y. et al., 2016). Bak also has estrogen-like effects (Lim et al., 2011; Weng et al., 2015). Estrogens are strong neuroprotectants that play a protective role in the oxidative stress, mitochondria damage, neuroinflammation, neurodegeneration, and other injury mechanisms in central nervous system (CNS) disease (Miller and Duckles, 2008; Villa et al., 2016; Engler-Chiurazzi et al., 2017). This evidence also suggests that Bak has a strong neuroprotective effect. However, no study about Bak’s direct effects on cerebrovascular disease has been reported previously.

In the present study, the protective effects of Bak against SAH injury have been demonstrated for the first time. The previously demonstrated antioxidative stress and anti-apoptosis effects of Bak have also been further validated in our study. Besides, it has also been confirmed in the present study that AMPK and Trx/TXNIP play a pivotal role in the antioxidative effect and neuroprotective effect of Bak. The protein kinase activity of AMPK plays a pivotal role in a variety of intracellular biological processes such as the growth of the cell, the metabolism of glucose and lipid, the oxidative stress, the maintenance of mitochondrial function and mitochondrial homeostasis, and the autophagy (Garcia and Shaw, 2017). The Trx system is a main intracellular antioxidant system, which detoxifies ROS and protects cells from oxidative damage (Cheng et al., 2011). While, TXNIP, as an intersection of oxidative stress and inflammasome activation, is essential in both of the oxidative damage and inflammatory injury (Zhou et al., 2010). Therefore, AMPK, Trx, and TXNIP might play a central role in the mechanism of Bak’s neuroprotective effect.

Our findings suggest that Bak has great potential for clinical application in the CNS system. However, the direct molecule target and its particular intracellular signal transduction mechanism of Bak still needs to be further revealed before Bak can be transformed for clinical use. The effects of Bak on other CNS diseases also need to be explored.

The present study was focused on the pretreatment effects of Bak. This route of drug administration is usually used to explore the preventive strategies of diseases. Thus, the findings in the present study can be applied in some clinical occasions. For example, patients are at a high risk of hemorrhage during the neurosurgical procedures dealing with intracranial diseases such as intracranial aneurysms and intracranial arteriovenous malformations. The pretreatment of Bak before surgery might improve patients’ tolerance to the possible hemorrhage injury. Besides, periprocedural aneurysm re-rupture is an extremely serious complication of endovascular treatment dealing with aneurysmal SAH. The mortality of patients with aneurysm re-rupture is as high as about 70% (Dmytriw et al., 2014; van Lieshout et al., 2019). The pretreatment of Bak might be a novel preventive therapeutic strategy against the re-rupture of cerebral aneurysms. In addition, patients with unruptured intracranial aneurysms and arteriovenous malformations are at a high risk of stroke when the lesion remains untreated. The preventive taking of Bak could be very beneficial for patients if the stroke occurs. Of course, urgent treatment after SAH is the most difficult problem in clinical practice. However, the effects of Bak post-treatment on SAH injury remain unclear. We will continue to explore this effect in the future.

Limitations still exist in the present study. The direct receptor and signal transduction pathways of Bak have not been fully explored. In subsequent studies, we will continue to explore the upstream and downstream regulators of TRX/TXNIP and AMPK. The effects of Bak on other injury mechanisms after SAH, such as neuroinflammation and autophagy, have not been observed. Thus, there is still a long way to go before the clinical application of Bak.

Another limitation is the measurement method of brain water content. The highest brain water contents in most of the studies using the same animal model did not reach 84%. However, the highest tested brain water content in the present study is about 85%. After careful reflection of our experimental protocol, we believe that the following factors might be the cause of this discrepancy. First, the sutures were advanced approximately 3 more millimeters after feeling the resistance to make sure the vascular was ruptured which might cause more serious traumatic brain injury than the operation in other studies. Second, the bloodstains and clots were not totally removed from the brain tissue before weighing in the present study. Third, the brains were handled on ice and might be stained with water released from the ice melt. Other unknown factors may also exist, which should be found out in our future study. Besides, the data in the present study cannot be used to compare the degree of cerebral edema at different time points post SAH, which is of great significance in the evolution process of SAH injury.

In conclusion, the present study confirms the protective effects of Bak against EBI after SAH in mice. These effects are mediated through alleviating BBB impairment, oxidative stress, and apoptosis by regulating Trx/TXNIP and AMPK phosphorylation. The powerful effects of Bak make it a promising novel drug for the treatment of EBI after SAH.

## Data Availability Statement

All datasets generated for this study are included in the article/supplementary material.

## Ethics Statement

The animal study was reviewed and approved by Ethics Committee of the Fourth Military Medical University.

## Author Contributions

YQ conducted this project and supported the research. HL designed the study and drafted the manuscript. HL, WG, HG, LZ, LY, and XL performed the experiments and acquired the primary data. JL, XW, and WC completed the statistics and interpreted the data. DF revised the manuscript.

## Funding

This study was supported by grants from the National Natural Science Foundation of China (81571215, 81630027).

## Conflict of Interest

The authors declare that the research was conducted in the absence of any commercial or financial relationships that could be construed as a potential conflict of interest.
